# High Positive Predictive Value of Multitarget Stool DNA After Aerodigestive Tract Radiotherapy

**DOI:** 10.1016/j.gastha.2022.05.002

**Published:** 2022-05-16

**Authors:** Derek W. Ebner, Jason D. Eckmann, Kelli N. Burger, Douglas W. Mahoney, Thomas J. Whitaker, Ivy A. Petersen, John B. Kisiel

**Affiliations:** 1 Division of Gastroenterology and Hepatology, Mayo Clinic, Rochester, Minnesota; 2 Division of Gastroenterology, Hepatology, and Nutrition, University of Minnesota, Minneapolis, Minnesota; 3 Division of Clinical Trials and Biostatistics, Mayo Clinic, Rochester, Minnesota; 4 Department of Radiation Physics, The University of Texas M. D. Anderson Cancer Center, Houston, Texas; 5 Department of Radiation Oncology, Mayo Clinic, Rochester, Minnesota

**Keywords:** Colorectal Cancer/Prevention and Control, Radiotherapy/Complications, Survivorship, DNA Neoplasm

## Abstract

**Background and Aims:**

Multitarget stool DNA (mt-sDNA) is approved for average-risk colorectal cancer screening; test performance in persons with prior radiation therapy (RT) has not been studied. RT can induce gastrointestinal bleeding and alter DNA methylation, which may affect mt-sDNA accuracy. Among patients previously treated with RT, we aimed to measure the positive predictive value (PPV) of mt-sDNA and compare these results to historical estimates of mt-sDNA PPV among average-risk patients.

**Methods:**

After institutional review board approval, we conducted a retrospective cohort study of a multisite academic and community-based practice. Patients with RT and subsequent mt-sDNA use during the study period (2014–2016) were identified. The findings at diagnostic colonoscopy were compared with published reports among average-risk patients. Nominal *P* values were generated by 2-tailed Fisher’s exact testing in comparisons of colorectal neoplasia (CRN) rates between groups.

**Results:**

There were 220 patients who had RT before mt-sDNA testing. RT was delivered along the aerodigestive tract in 108 patients. Mt-sDNA tests were positive in 45 of 220 patients (20%), and colonoscopy findings were available for 42; 31 of 42 patients (74%) had CRN. PPV by mt-sDNA was similar when stratified by site of prior RT (along vs outside the aerodigestive tract; *P* = 1.00). Detection of advanced CRN (36%) was nominally higher than previously published retrospective (27%) and prospective (20%) studies. The median time from the start of RT to mt-sDNA use was 7 (interquartile range, 3–14) years.

**Conclusion:**

With a test positivity rate and PPV for CRN similar to reports among average-risk patients, prior RT does not appear to adversely affect mt-sDNA performance.

## Introduction

Persons with a history of cancer can develop long-term complications from prior cancer treatment and have risk for developing additional primary malignancies. Evidence for the prevention of cancers among cancer survivors are lacking.^1^ In addition, preventive health care for cancer patients may not receive sufficient emphasis and has historically been underutilized.^2^^,^^3^ However, survivors are likely to adhere to cancer screening services when accessible.^4^^,^^5^ Long-term care of cancer survivors is of particular importance realizing that there are nearly 17 million current cancer survivors in the United States and more than 22 million are estimated by 2030.^6^ Despite this growing number, cancer survivor–specific guidelines have only been developed for breast cancer^7^ and for cancers of childhood.^8^

Colon cancer prevention is hampered by several challenges, well described in the average-risk population. Despite remaining the second leading cause of cancer-related deaths among men and women combined,^9^ one-third of all eligible adults are unscreened for colorectal cancer (CRC).^10^ Among the general population, adherence to CRC screening is improved through utilization of noninvasive screening, which will likely shape the future of CRC screening.^11^ Of the United States Preventive Services Task Force recommendations for CRC screening, the annual fecal immunochemical test (FIT) and multitargeted stool DNA (mt-sDNA) test every 1–3 years are the most commonly used stool-based modalities.^12^, ^13^, ^14^ However, the optimal approach to CRC screening in cancer survivors is uncertain, particularly for those who have received cancer radiation therapy (RT) to the gastrointestinal tract.

RT can induce a broad spectrum of complications, such as secondary malignancy, fistula, fibrosis, and bleeding.^15^ For example, in reference to the general population, the treatment effects for Hodgkin lymphoma are associated with increased risk for colorectal advanced neoplasia (AN), including advanced sessile serrated polyps.^16^ RT to the gastrointestinal tract also raises the risks of complications during endoscopy, including screening colonoscopy,^17^ and these risks have increased both patient and provider interest in noninvasive, stool-based CRC screening for cancer survivors. However, RT may have lasting effects on gastrointestinal anatomy and physiology that could theoretically adversely affect the accuracy of stool-based testing. Radiation-induced gastrointestinal bleeding could lead to artifactual occult bleeding and subsequent unnecessary use of diagnostic colonoscopy. Mt-sDNA detects fecal hemoglobin through an immunoassay and additionally quantifies tumor-associated DNA markers (methylated *BMP3* and *NDRG4*, mutant *KRAS*, and *β-actin*) and reports a positive/negative result based upon a validated algorithm.^18^ The inclusion of DNA markers contributes to greater sensitivity for CRC and other AN when compared with FIT in average-risk patients.^19^, ^20^, ^21^ However, radiation directly alters DNA methylation both acutely^22^ and during cellular regrowth needed for tissue healing, which could theoretically lead to additional false-positive mt-sDNA testing.^23^

Despite the concern for false-positive stool-based testing in those with prior RT, there is a paucity of literature to support this or to further aid in decision-making for noninvasive CRC screening. To address this knowledge gap, we aimed to describe the use of mt-sDNA testing in those with a history of RT stratified by radiation site (aerodigestive vs nonaerodigestive tract) and measure the positive predictive value (PPV) at diagnostic colonoscopy. These findings were then compared with previously published reports of mt-sDNA performance among patients at average risk for CRC, without prior RT, to infer whether RT alters the PPV of mt-sDNA.

## Materials and Methods

A multicenter retrospective cohort study was conducted after institutional review board approval. Data were collected from patients who had undergone mt-sDNA testing between September 25, 2014, and December 30, 2016, at any of the Mayo Clinic referral centers (Minnesota, Arizona, and Florida) or surrounding community practices of the Mayo Clinic Health System (Minnesota, Wisconsin, and Iowa).

### Cohort Identification

The Unified Data Platform of Mayo Clinic maintains an electronic search engine (advanced cohort explorer) to query patient data across the collective Mayo Clinic enterprise. This clinical data repository contains clinic notes that can be searched by natural language processing as well as functionality to search fixed diagnosis or billing codes. Because of the changes in the electronic medical record system in the Mayo Clinic Enterprise, information on exposure to RT could only be queried from the Unified Data Platform through the calendar year 2016, using the following validated search heuristic.

**Figure 1:**
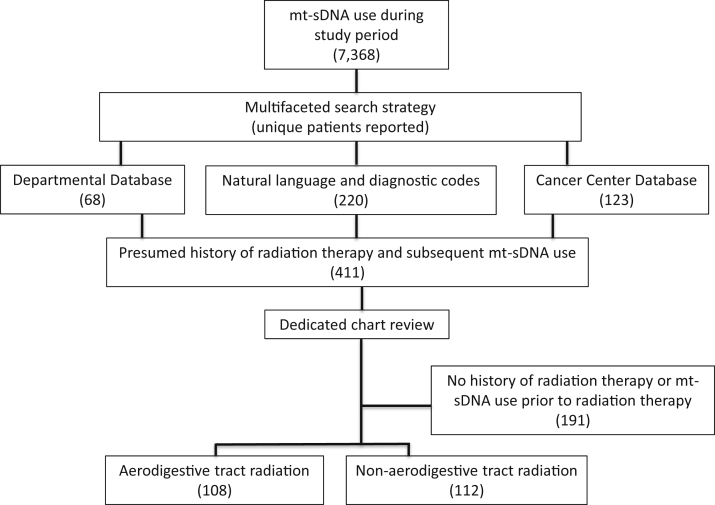
Study flow diagram of cohort creation.

First, mt-sDNA use and test results were identified through diagnostic and procedure billing codes. Then, a multifaceted search strategy was used to explore the mt-sDNA cohort to identify those with a potential history of RT or cancer whose medical records would then undergo dedicated review (Figure 1). The search strategy included a query of available diagnostic codes (HIC, SNOMED, and ICD-9/10) relating to cancer and RT available through the advanced cohort explorer program and a free text search for “cGy” or “radiotherapy” or “brachytherapy” or “external beam.” The entire mt-sDNA group was also compared with 3 separate databases that are maintained by the Department of Radiation Oncology to identify those with current or former RT plans. Finally, the entire mt-sDNA group was referenced with the Cancer Center database for the entire Mayo Clinic enterprise to identify patients with any cancer history. Patients with radiation exposure after mt-sDNA testing were excluded; the remaining patients identified from these queries underwent dedicated chart review to establish the final cohort.

### Data Collection for Post-RT Cohort Screened by mt-sDNA

**Figure 2:**
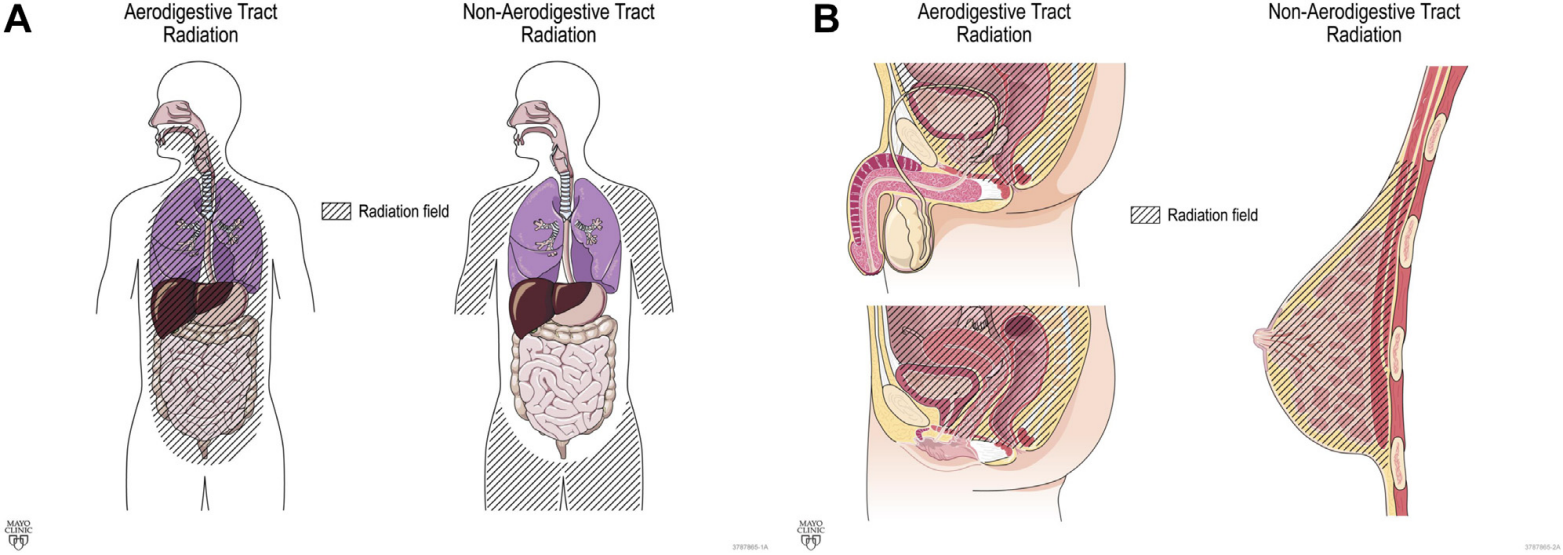
Classification of radiation field (A) Coronal and (B) Sagittal.

A trained data extraction team verified RT history, date of mt-sDNA use, and post-mt-sDNA colonoscopy results through individual chart review. Abstracted patient characteristics included sex, age, and tobacco use. History of colonoscopy before mt-sDNA was recorded. For those with positive mt-sDNA who underwent diagnostic colonoscopy, the presence of colorectal neoplasia (CRN) neoplasia, CRN architecture (adenoma [tubular, tubulovillous, or villous] or sessile serrated polyps), dysplasia grade (high vs low), CRN size (diameter, in millimeter), and/or CRC was abstracted. Hyperplastic polyps regardless of size were not counted as neoplasia. Specific clinical variables that would classically exclude a patient from approved use of mt-sDNA (history of high-risk CRN, digestive cancer, inflammatory bowel disease, polyposis syndromes, family history of colorectal cancer ≤61 years old, overt rectal bleeding [within 90 days of mt-sDNA testing], positive fecal blood testing within 6 months of mt-sDNA use, iron deficiency anemia within 90 days of mt-sDNA use, and/or aerodigestive cancer within 5 years of mt-sDNA use) were noted, but these patients were not excluded from the primary analysis. For the historical malignancy treated by RT, the stage was recorded from clinical notes or pathology reports generated during the patient’s care but was not updated to the current American Joint Committee on Cancer staging guidelines. If a patient had a history of more than one primary cancer, the initial malignancy was abstracted. The most recent RT course start and end dates were recorded, and exposure defined by radiation field (head/neck, thoracic, upper abdomen, mantle, aortic, pelvis/paraaortic, pelvic, prostate, and vaginal cuff) and type (external beam, brachytherapy, or both). The RT exposed mt-sDNA cohort was grouped by radiation site (aerodigestive vs nonaerodigestive tract; Figure 2). All data were entered into a secure online database built using REDCap (Vanderbilt University, Nashville, TN).

### Historical Data for Average-Risk CRC Screening by mt-sDNA

The test positivity rate of mt-sDNA and findings at diagnostic colonoscopy in those without a history of RT was compared with 2 published reports of mt-sDNA use among independent patients at average risk for CRC. The first was the pivotal trial for mt-sDNA Food and Drug Administration (FDA) approval; the “DeeP-C” study (ClinicalTrials.gov Identifier: NCT01397747),^19^ a prospective cross-sectional cohort observational trial designed to estimate mt-sDNA sensitivity and specificity, in comparison to blinded FIT and criterion standard colonoscopy. The second comparative study was a retrospective review of mt-sDNA testing across all Mayo Clinic sites from October 1, 2014, through December 31, 2017.^24^ As the RT cohort may have met inclusion criteria within this comparative study, the final RT cohort was cross-referenced with this published report and when present were included only in the RT cohort to ensure comparisons between unique patients.

### Test Positivity Rate Stratified by Sex and Age

As a reference population of both average and increased risk individuals for CRC, we used the Unified Data Platform for the Mayo Clinic enterprise to tally positive and negative mt-sDNA results between 2014 and 2019. This cohort was examined solely to measure the number of positive tests among all tests completed and to ensure that no patients in the 2014–2016 RT exposed cohort were included in this reference group of unique individuals. This reference population was then matched by age and sex to the RT cohort to compare mt-sDNA positivity rates.

### Statistical Analysis

Patient characteristics of the radiation-treated, mt-sDNA-tested cohort were compared with the 2 historical average-risk cohorts using the Wilcoxon rank-sum test for continuous variables (summarized as a median with corresponding 25th and 75th percentiles), whereas proportions were compared by the Fisher’s exact test. PPV was estimated as the number of positive findings relative to the total number of patients undergoing diagnostic colonoscopy with corresponding 95% confidence intervals (CIs). The Chi-square goodness-of-fit test with 3 degrees of freedom was used to compare the observed number of positive mt-sDNA results within the total radiation cohort relative to the expected number of positive mt-sDNA results based on an age- (grouped in decades of 50–59, 60–69, 70–79, and 80+) and sex-matched cohort of patients (independent of CRC risk) that underwent mt-sDNA testing at Mayo Clinic between 2014 and 2019. As the effect size on PPV from prior RT exposure was not known, an *a priori* power calculation was not performed, and statistical comparisons are reported with nominal *P* values.

## Results

### Study Population

During the study period, 7368 patients had undergone mt-sDNA testing across all Mayo Clinic sites. After this group was cross-referenced to established databases and registries and searched by natural langue processing and diagnostic codes, 411 unique patients were identified and underwent individual chart review. From these, 220 patients met inclusion criteria (Figure 1). These 220 patients were then cross-referenced with the cohort described by Eckmann et al^24^; the 46 overlapping patients were analyzed only as members of the RT cohort.

The median age of the post-RT mt-sDNA cohort was 71 (interquartile range [IQR], 64–77) years, and most were female (149/220, 68%); both were significantly greater for the RT mt-sDNA cohort when compared with prior cohorts of mt-sDNA-tested patients (Table 1); the RT cohort was also less racially diverse in comparison to the DeeP-C study cohort, which enrolled patients from more than 90 North American sites.^19^

**Table 1:** Patient Characteristics Among Those Screened by mt-sDNA With or Without a History of Radiation Therapy

Patient characteristic	Radiation therapy (RT) mt-sDNA (N = 220)	Eckmann et al^d^ (n = 1523)	Imperiale et al (n = 9989)	*P* value RT mt-sDNA compared to:	
				Eckmann et al^d^	Imperiale et al
Median age, y (IQR) or [SD]	71 (64–77)	67 (61–73)	64.2 [8.41]	<.0001	<.0001
Men, n (%)	71 (32)	613 (40)	4625 (46)	.03	<.0001
White race, n (%)	207 (95)^a^	1463 (96)	8392 (84)	.46	<.0001
Current or former tobacco, n (%)	97 (44)	750 (50)^b^	4492 (45)^c^	.13	.84

a Race missing in 2 patients.

b Tobacco history was missing for 12 patients.

c Tobacco history was reported for the baseline group (n = 10,023); separate reporting of the evaluable group was not reported.

d Among this published cohort, patients with history of radiation therapy and subsequent mt-sDNA, use were removed from the Eckmann cohort and included only in the radiation therapy group.

### Radiation and Prior Colonoscopy History

**Figure 3:**
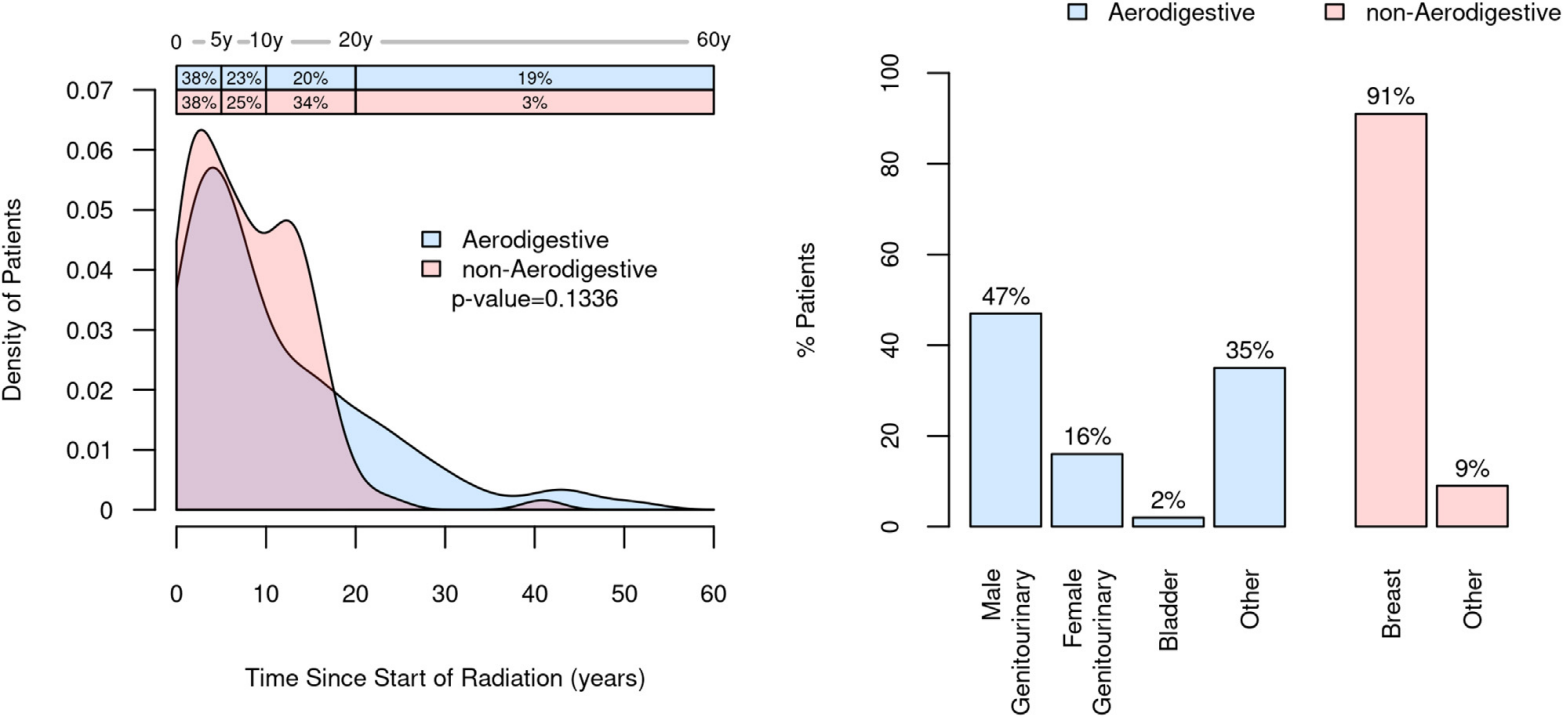
Distribution of the radiation field along the aerodigestive and nonaerodigestive tracts and time from the initiation of radiation therapy to mt-sDNA testing.

For the RT cohort, the field of therapy had been directed to include the aerodigestive tract for 108 of 220 (49%), and of those, cancers of male genitourinary tract were the most common (51/108, 47%). For nonaerodigestive RT, breast cancer was the most common indication (102/112, 91%; Figure 3). Further cancer characteristics are provided (Table A1). For this entire cohort, external beam therapy was most common (202/220, 92%), and 18 (8%) had brachytherapy with or without external beam therapy. The median time from the start of RT to the day of mt-sDNA testing was 7 (IQR, 3–14) years for the entire cohort. There were no significant differences in time from RT to mt-sDNA testing when stratified by radiation field (Figure 3).

Of the 220 patients, 144 (65%) had a history of colonoscopy, and only 12 patients had CRN detected at prior colonoscopy; 3 of whom had high-risk CRN (polyp size ≥10 mm or 5–10 adenoma <10 mm).^25^

### CRN in RT Cohort

For patients who had mt-sDNA testing and prior RT exposure, the test positivity rate was 45 of 220 (20% [95% CI, 15%–26%]). Of the 45 patients with a +mt-sDNA, 42 (93%) underwent diagnostic colonoscopy. The mt-sDNA test positivity rate after RT was greater in comparison to average-risk patients without RT from our institution^24^ although the rate of colonoscopy compliance was similar (Table 2).

**Table 2:** Colorectal Neoplastic Findings in Those Having Follow-Up Colonoscopy for Positive mt-sDNA Test With or Without Prior Radiation Therapy

Finding	Radiation therapy (RT) mt-sDNA (n = 220)	Eckmann et al^d^ (n = 16,249)	Imperiale et al (n = 9989)	*P* value RT mt-sDNA compared to:	
				Eckmann et al^d^	Imperiale et al
Positive mT-sDNA, n (%)	45 (20)	2280 (14)	1612 (16)	.01	.10
Colonoscopy compliance^a^, n (%)	42 (93)	1523 (86)^b^	9989 (100)	.26	N/A
Any colorectal neoplasia, n (%)	31 (74)	1020 (67)	879 (55)	.41	<.0001
Colorectal cancer, n (%)	0 (0)	14 (1)	60 (4)	1.00	.40
Advanced colorectal neoplasia^c^, n (%)	15 (38)	415 (27)	321 (20)	.22	.02
Nonadvanced neoplasia, n (%)	16 (38)	591 (39)	498 (31)	1.00	.32
No neoplasia, n (%)	11 (26)	503 (33)	733 (45)	.41	.02
≥3 polyps^e^ <10 mm, n (%)	7 (18)	154 (10)	Not reported	.19	N/A
1–2 polyps 6–9 mm, n (%)	5 (12)	163 (11)	Not reported	.80	N/A
1–2 polyps ≤5 mm, n (%)	4 (10)	274 (18)	Not reported	.22	N/A

a One additional patient was reported to have undergone post-mt-sDNA, colonoscopy at another center but this could not be confirmed.

b Test positive analysis excluded 220 patients that were at increased risk for colorectal cancer and 297 patients that denied consent for research or did not meet inclusion criteria.

c Adenoma/sessile serrated polyps ≥1 cm or with high grade dysplasia or villous elements.

d Among this published cohort, patients with history of radiation therapy and subsequent mt-sDNA, use were removed and included only in the RT, group.

e Polyp refers to tubular adenoma or sessile serrated adenoma; Eckmann paper reported CRC, with advanced neoplasia.

At colonoscopy after +mt-sDNA among the RT cohort, 31 of 42 (74% [95% CI, 58%–86%]) had any CRN, and 15 of 42 (36% [95% CI, 22%–52%]) had advanced CRN, both greater in comparison to the DeeP-C study (55% and 20%, respectively).^19^ Additional subcategorization of the highest graded neoplasms at post-mt-sDNA colonoscopy is shown (Table 2). The PPV for CRN was not impacted by RT delivery along the aerodigestive tract or outside of the aerodigestive tract (74% for both; *P* = 1.00).

### CRN in Post-RT Patients at Average CRC Risk

Of the 220 patients with radiation history, 25 patients had criteria that should have excluded the use of mt-sDNA (personal history of high-risk CRN, IBD, overt rectal bleeding, and family history of CRC ≤61 years old). Among the 25 patients with standard exclusion criteria, 8 (32% [95% CI, 15%–54%]) had a +mt-sDNA. Among 195 patients with no FDA labeling exclusion criteria for mt-sDNA use, 37 (19% [95% CI, 14%–25%]) had a +mt-sDNA vs 20% in the overall positivity rate in an age–sex balance cohort of non-RT patients (*P* = .1154). For patients without FDA labeling exclusions for mt-sDNA and post-mt-sDNA colonoscopy, PPV for CRN was 28 of 34 (82% [95% CI, 65%–93%]), and for those with mt-sDNA exclusions the PPV was 3 of 8 (38% [95% CI, 9%–76%]; *P* = .0195).

**Figure 4:**
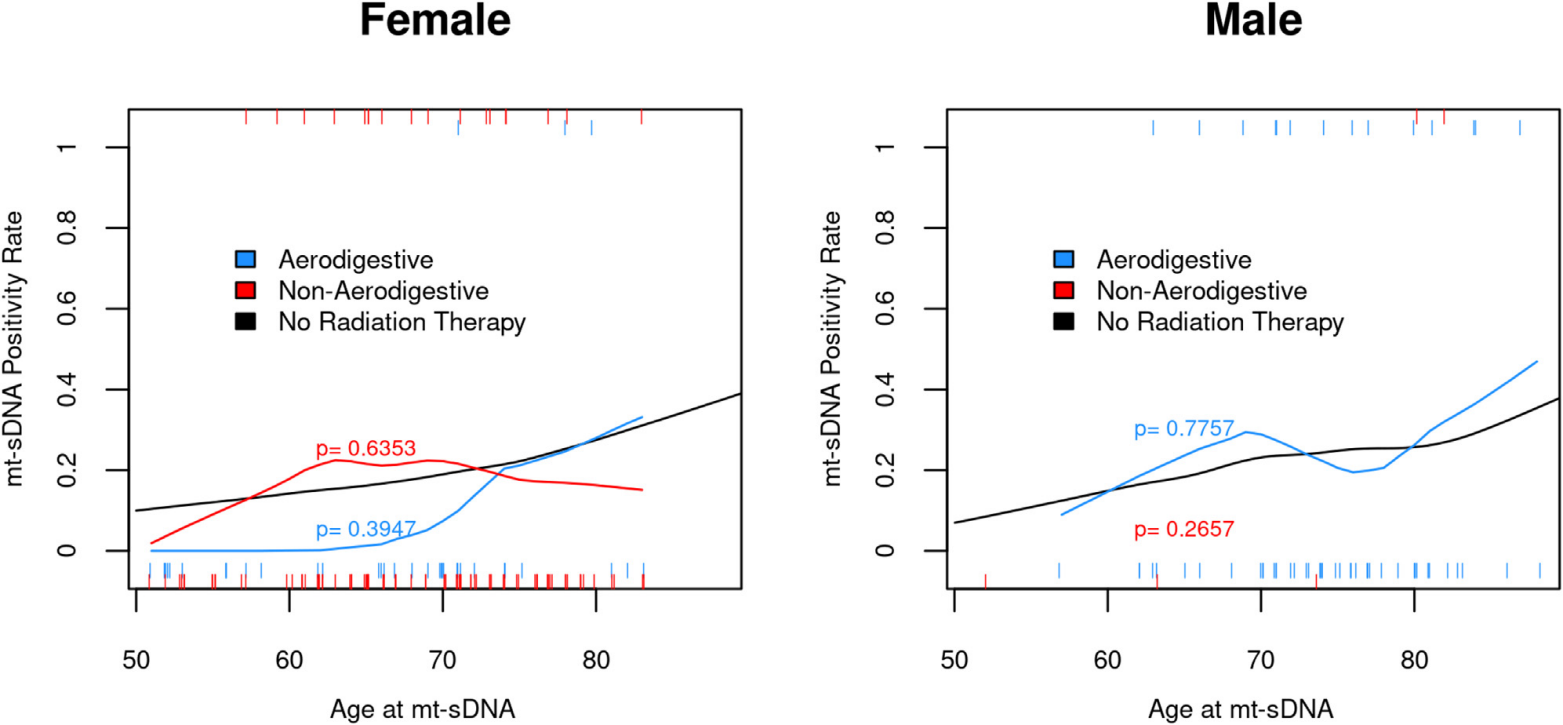
Mt-sDNA positivity rate is not influenced by radiation therapy after stratification for age and sex compared with average-risk patients without radiation therapy.

Among patients without labeling exclusions with RT along the aerodigestive tract, there were 38 women with aerodigestive tract radiation with 3 (8%) +mt-sDNA findings compared with the expected number of findings of 6.8 +mt-sDNA findings based on an age-matched female cohort of (*P* = .3947). Similarly, there were 97 women with nonaerodigestive RT with 18 (13%) +mt-sDNA findings compared with the expected number of 18.1 (*P* = .6353). For 55 men receiving RT along the aerodigestive tract, 14 (25%) had +mt-sDNA findings compared with the expected number of findings of 13.3 (*P* = .7757). There were 5 men with RT at non-aerodigestive sites with 2 (40%) +mt-sDNA findings compared with the expected number of 1.1 findings (*P* = .2657). Overall, when stratified by sex and age, the mt-sDNA positivity rate is similar among patients without RT history vs the RT cohort (Figure 4).

## Discussion

Mt-sDNA test performance in patients who have had RT appears similar to or greater than that seen in previous studies of the general average-risk population. A high PPV was observed despite the concern of RT-induced gastrointestinal bleeding^15^ and alteration in DNA methylation.^22^^,^^23^ Had RT adversely influenced mt-sDNA performance, a lower PPV would have been anticipated. The high PPV of mt-sDNA for CRN was independent of radiation field (aerodigestive [74%] vs nonaerodigestive tract [74%, *P* = 1.00]) and nominally higher than the PPV reported among historical cohorts of average-risk patients without RT history. When excluding RT patients who had FDA labeling exclusions for the use of mt-sDNA, the PPV increased to 82%. This high rate is supported by the findings of Ykema et al^26^ who are the only investigators to prospectively compare the use of FIT and mt-sDNA among Hodgkin lymphoma survivors with colonoscopy as the reference standard. This study was conducted at 4 Dutch centers, and among the 2 noninvasive tests, the PPV for AN ranged from 41% to 63%; mt-sDNA had the greatest sensitivity (68%) in comparison to FIT (37%).

Real-world mt-sDNA test use in the general population provides valuable context for the observations in this study. In the DeeP-C study, colonoscopists were blinded to mt-sDNA results.^19^ Colonoscopy yield was later studied for a subset of this same group of colonoscopists when aware (unblinded) of the +mt-sDNA result, and significantly more polyps were found.^27^ This change in performance was thought to result, in part, to greater colonoscopist attention as withdrawal times were significantly longer among those unblinded. This phenomenon may explain the greater CRN and advanced CRN detection among the RT cohort by unblinded colonoscopists evaluating +mt-sDNA results, compared with the DeeP-C study. Advanced CRN was found for 15 (7%) of all 220 patients screened by mt-sDNA in the RT cohort, which is very similar to what would be expected for advanced CRN yield by screening colonoscopy (7.6% in DeeP-C). This high rate of advanced CRN detection by mt-sDNA may similarly have been influenced by unblinded diagnostic colonoscopy. In addition, the higher observed PPV for advanced CRN is also possibly a result of increased CRN risk after undergoing RT itself,^28^, ^29^, ^30^, ^31^, ^32^ other cancer treatments,^33^, ^34^, ^35^ or for patients having a history of extracolonic cancer.^36^^,^^37^

If a history of RT accelerates the natural history of CRN, testing at more frequent intervals may be required to optimize screening effectiveness. Although mt-sDNA test performance after RT appears comparable to that of the general population, the optimal and most cost-effective interval of mt-sDNA testing in patients with prior RT has yet to be determined. The testing interval for mt-sDNA in average-risk patients is based on the Archimedes model (Archimedes Inc, San Francisco, CA) in which the risk factors for CRC include age, gender, race, and body mass index. Although annual mt-sDNA performed similarly to colonoscopy; mt-sDNA testing at an interval of 3 years had greater cost-effectiveness and patient adherence.^38^

Although informative, this study is limited by the retrospective design. Therefore, we had no control of variables, such as cancer type, stage, and cumulative radiation exposure. However, it is reassuring that when compared with real-life observations across the same centers by Eckmann et al^24^ and a prospectively enrolled cohort where the majority of patients were enrolled outside our centers,^19^^,^^26^ that mt-sDNA PPV for CRN and advanced CRN were comparable. Furthermore, as a study of real-life clinical practice, patients with a negative mt-sDNA did not undergo colonoscopy. As such, we are not able to calculate the negative predictive value. Our study design is also unable to determine whether RT induces hypomethylation to *BMP3* and *NDRG4* genomic loci, ultimately resulting in a false-negative mt-sDNA. With these limitations in mind, we conducted further data extraction post-hoc for the 175 patients who had a negative mt-sDNA test between 2014 and 2016. Among this group, we reviewed the electronic medical record through March 1, 2022. For 105 of 175 (60%) of the patients, there was subsequent mt-sDNA or colonoscopy use. Subsequent colonoscopy was conducted for 25 of 105 (24%) patients, whereas mt-sDNA was pursued by 80 of 105 (76%). For those with subsequent mt-sDNA use, the test positivity rate was 27.5% (22/80 [95% CI, 18%–39%]), which is similar to 45 of 220 (20% [95% CI, 15%–26%]) to first round testing. The median time from first round testing to the second mt-sDNA (N = 80) was 3.4 years (IQR, 3.1–4.0). Diagnostic colonoscopy was done for 20 of 22 patients, neoplasia was found for 11 of 20 patients (55% [95% CI, 32%–77%]), and 35% (7/20 [95% CI, 15%–59%]) had AN compared with 31 of 42 (74% [95% CI 58%–86%]) and 15 of 42 (36% [95% CI, 22%–52%]) for first round testing. For the 25 patients with colonoscopy use after prior negative mt-sDNA, the median time was 3.1 years (IQR, 1.7–3.3), and neoplasia was diagnosed for 9 of 25 (36% [95%CI, 18–57]) of which 16% (4/25 [95% CI, 5%–36%]) was AN. Thus, the test positive rate and the PPV of mt-sDNA on subsequent screening appear similar to the first round of screening, lowering the possibility of missed screen relevant lesions on the first round. Importantly, there were no symptomatic or screen-detected colorectal cancers diagnosed after prior negative mt-sDNA. There are other notable study limitations. Although the time from RT to mt-sDNA was similar among aerodigestive vs nonaerodigestive radiation exposure, there was a wide distribution of time from RT to mt-sDNA testing; therefore, this cohort may not be representative of those who are tested by mt-sDNA beyond 20 years from RT start time. We note that radiobiologic models as well as clinical data suggest that radiation carcinogenesis is a stochastic effect, not dose dependent, as such we suspect little utility in further qualifying the influence of radiation dose and the performance of mt-sDNA.^39^^,^^40^ Generalizability of our findings may be limited, as the overall cohort had more women than men, but our additional analysis of mt-sDNA comparing outcomes by sex and age among patients with and without RT found no differences (Figure 4). The higher prevalence of women may be related to adherence to breast cancer survivor guidelines.^7^ Finally, the small subset of patients in the cohort with brachytherapy may be perceived as a study limitation. However, brachytherapy is only used in select situations and is anticipated to comprise a smaller proportion of RT.

Overall, mt-sDNA testing appears to be a promising method for colorectal cancer screening among those with a history of RT. Given the high PPV of mt-sDNA for both any CRN and advanced CRN among those with or without prior RT, a positive test should be promptly followed by diagnostic colonoscopy.

## Supplementary Materials

Table A1
