# Supplementary material for: High Positive Predictive Value of Multitarget Stool DNA After Aerodigestive Tract Radiotherapy
Source: Gastro Hep Adv. 2022 May 16;1(5):746–54. doi: 10.1016/j.gastha.2022.05.002 (PMC9481191; doi:10.1016/j.gastha.2022.05.002)
Supplement: Table A1 [file mmc1.docx]

Supplemental material 1: Indication for radiation therapy by cancer type and region among those with radiation therapy followed by multi-target stool DNA (mt-sDNA) testing

| **Cancer Indication, n (%)** |
| --- |
| **Aerodigestive Tract, 108 (100)** |
| Prostate, 49 (45) |
| Testicular, 2 (2) |
| Lymphoma, 13 (12) |
| Leukemia, 3 (3) |
| Head and neck, 12 (11) |
| Endometrial, 9 (8) |
| Cervix or vagina, 6 (5.5) |
| Ovarian, 2 (2) |
| Lung, 6 (5.5) |
| Bladder, 2 (2) |
| Renal or retroperitoneal, 1 (1) |
| Ampulla/Pancreas, 1 (1) |
| Esophagus, 1 (1) |
| Malignant thymoma, 1 (1) |
|  |
| **Non-Aerodigestive Tract, 112 (100)** |
| Breast, 102 (91) |
| Bone (extremity, spine), 6 (5) |
| Head, 4 (4) |
